# Diverse dystonin gene mutations cause distinct patterns of *Dst* isoform deficiency and phenotypic heterogeneity in *Dystonia musculorum* mice

**DOI:** 10.1242/dmm.041608

**Published:** 2020-05-21

**Authors:** Nozomu Yoshioka, Yudai Kabata, Momona Kuriyama, Norihisa Bizen, Li Zhou, Dang M. Tran, Masato Yano, Atsushi Yoshiki, Tatsuo Ushiki, Thomas J. Sproule, Riichiro Abe, Hirohide Takebayashi

**Affiliations:** 1 Division of Neurobiology and Anatomy, Graduate School of Medical and Dental Sciences, Niigata University, Niigata 951-8510, Japan; 2 Transdiciplinary Research Programs, Niigata University, Niigata 950-2181, Japan; 3 Division of Dermatology, Graduate School of Medical and Dental Sciences, Niigata University, Niigata 951-8510, Japan; 4 Center for Coordination of Research Facilities, Niigata University, Niigata 951-8510, Japan; 5 RIKEN BioResource Research Center, Tsukuba 305-0074, Japan; 6 Division of Microscopic Anatomy, Graduate School of Medical and Dental Sciences, Niigata University, Niigata 951-8510, Japan; 7 The Jackson Laboratory, Bar Harbor, ME 04609, USA

**Keywords:** *Dystonia musculorum* mice, Dystonin/Bpag1, Hemidesmosome, Neuropathy

## Abstract

Loss-of-function mutations in dystonin (*DST*) can cause hereditary sensory and autonomic neuropathy type 6 (HSAN-VI) or epidermolysis bullosa simplex (EBS). Recently, *DST*-related diseases were recognized to be more complex than previously thought because a patient exhibited both neurological and skin manifestations, whereas others display only one or the other. A single *DST* locus produces at least three major *DST* isoforms: *DST-a* (neuronal isoform), *DST-b* (muscular isoform) and *DST-e* (epithelial isoform). *Dystonia musculorum* (*dt*) mice, which have mutations in *Dst*, were originally identified as spontaneous mutants displaying neurological phenotypes. To reveal the mechanisms underlying the phenotypic heterogeneity of *DST*-related diseases, we investigated two mutant strains with different mutations: a spontaneous *Dst* mutant (*Dst^dt-23Rbrc^* mice) and a gene-trap mutant (*Dst^Gt^* mice). The *Dst^dt-23Rbrc^* allele possesses a nonsense mutation in an exon shared by all *Dst* isoforms. The *Dst^Gt^* allele is predicted to inactivate *Dst-a* and *Dst-b* isoforms but not *Dst-e*. There was a decrease in the levels of *Dst-a* mRNA in the neural tissue of both *Dst^dt-23Rbrc^* and *Dst^Gt^* homozygotes. Loss of sensory and autonomic nerve ends in the skin was observed in both *Dst^dt-23Rbrc^* and *Dst^Gt^* mice at postnatal stages. In contrast, *Dst-e* mRNA expression was reduced in the skin of *Dst^dt-23Rbrc^* mice but not in *Dst^Gt^* mice. Expression levels of Dst proteins in neural and cutaneous tissues correlated with *Dst* mRNAs. Because *Dst-e* encodes a structural protein in hemidesmosomes (HDs), we performed transmission electron microscopy. Lack of inner plaques and loss of keratin filament invasions underneath the HDs were observed in the basal keratinocytes of *Dst^dt-23Rbrc^* mice but not in those of *Dst^Gt^* mice; thus, the distinct phenotype of the skin of *Dst^dt-23Rbrc^* mice could be because of failure of Dst-e expression. These results indicate that distinct mutations within the *Dst* locus can cause different loss-of-function patterns among *Dst* isoforms, which accounts for the heterogeneous neural and skin phenotypes in *dt* mice and *DST*-related diseases.

## Abstract (precis)

**Summary:** This paper describes the mechanism by which diverse dystonin gene mutations result in phenotypic heterogeneity in neural and cutaneous tissues of *Dystonia musculorum* mice.

## INTRODUCTION

*Dystonia musculorum* (*dt*) mice were originally characterized as spontaneously occurring mutants that display severe degeneration of sensory neurons in the dorsal root ganglion (DRG) and progressive motor symptoms such as dystonia-like movements and ataxia (Duchen et al., 1964). The causative gene for *dt* mice is dystonin (*Dst*), also known as bullous pemphigoid antigen1 (*Bpag1*) (Brown et al., 1995; Guo et al., 1995). Because of the large size of the *Dst* locus (encoding over 100 exons) and obvious motor phenotypes, many untargeted *Dst* mutant mouse strains have been established, including many spontaneously occurring mutants [*Dst^dt^* (Duchen et al., 1964); *Dst^dt-alb^* (Messer and Strominger, 1980); *dt^Frk^* (Pool et al., 2005); *Dst^dt-23Rbrc^* (Horie et al., 2016); *dt-MP* (Seehusen et al., 2016)], a transgene insertion-induced mutant [*Dst^Tg4^* (Kothary et al., 1988)] and chemically induced mutants [*Dst^dt-33J^* (MGI: 1889074); *Dst^dt-36J^* (MGI: 2681971)]. Several mutants were also intentionally generated, including a gene-targeting knockout [*Dst^tm1Efu^* (Guo et al., 1995)], a gene-trap mutant [*Dst^Gt(E182H05)^* (Horie et al., 2014)] and nuclease-mediated mutants [*Dst^em13Dcr^* (MGI: 6258920); *Dst^em14Dcr^* (MGI: 6258923)]. However, a limited number of these genomic DNA mutations have been identified precisely, including *Dst^Tg4^*, *Dst^dt-alb^*, *Dst^dt-23Rbrc^*, *dt-MP*, *Dst^tm1Efu^* and *Dst^Gt(E182H05)^*. Different transcription start sites and alternative splicing produce several *Dst* isoforms in a tissue-selective manner. The three major *Dst* isoforms that are predominantly expressed in neural, muscular and epidermal tissues are *Dst-a/Bpag1a*, *Dst-b/Bpag1b* and *Dst-e/Bpag1e*, respectively, each of which has its own distinct cytoskeleton-binding domains (Leung et al., 2001; Horie et al., 2017). All known Dst isoforms include a plakin domain and belong to the plakin family of proteins. The Dst-e isoform, also known as BPAG1 or BP230, localizes to the inner plaque of hemidesmosomes (HDs), adhesion complexes between basal epithelial cells in the epidermis and basement membranes (Nievers et al., 1999; Walko et al., 2015). Since *Dst* knockout (*Dst^tm1Efu^*) mice display structural abnormalities of HDs, subepithelial bulla and retardation of wound healing, Dst-e protein is also considered to maintain epidermal integrity (Guo et al., 1995). Further support for this idea is that the Dst-e isoform is well known to be a self-antigen in bullous pemphigoid (Künzli et al., 2016), a distinctly different disease that results in symptoms strikingly similar to those from epidermolysis bullosa simplex (EBS).

Loss-of-function mutations in human *DST* are related to hereditary sensory and autonomic neuropathy type VI (HSAN-VI) and/or the skin blistering disease, EBS. Patients with HSAN-VI harbor mutations in *DST-a* and suffer from reduced sensation to pain, touch, vibration, and autonomic disturbances such as reduced sweating, absent pupillary light reflexes, cardiovascular dysregulation and gastrointestinal dysmotility (Edvardson et al., 2012; Manganelli et al., 2017; Fortugno et al., 2019). Patients with EBS carrying loss-of-function mutations in *DST-e* display skin blisters (Groves et al., 2010; Takeichi et al., 2015; He et al., 2017; Turcan et al., 2017). Because the *DST* locus generates several *DST* isoforms, it is assumed that the heterogeneity of *DST*-related diseases may be due to mutations that affect a single transcript or multiple transcripts among DST isoforms. Although EBS and HSAN-VI have been described as distinct pathologies in most cases, a recent report describes a mixed case: a patient harboring the compound heterozygous mutation within the specific exon in *DST-a* and an exon common to both *DST-a* and *DST-e*, suffering from both neurological and skin manifestations (Cappuccio et al., 2017). It has also been reported that patients with *DST-a2*-specific mutations show late-onset HSAN-VI (Manganelli et al., 2017). Therefore, to understand the diverse spectrum of *DST*-related diseases, it is important to investigate phenotypic heterogeneity in *dt* strains.

In this study, we have characterized the deficiencies for *Dst* isoforms and phenotypic heterogeneity in two *Dst* mutants: *Dst^dt-23Rbrc^* and *Dst^Gt^* mice. In the *Dst^dt-23Rbrc^* allele, a nonsense mutation was identified in the plakin domain, which is a common domain in all *Dst* isoforms (Horie et al., 2016). In the *Dst^Gt^* allele, the gene-trap construct was inserted within the coding region of the actin-binding domain (ABD) at the N-terminus shared by *Dst-a* and *Dst-b* (Horie et al., 2014), but not contained in *Dst-e*. Our results indicate that diverse mutations within the *Dst* locus cause distinct loss-of-function patterns among *Dst* isoforms and distinct pathological outcomes in neural and cutaneous tissues. These results demonstrate phenotypic heterogeneity in *dt* mice and *DST*-related diseases and substantiate the necessity for additional studies of *Dst* mutations.

## RESULTS

### Expression patterns of *Dst* isoforms in *Dst^dt-23Rbrc^* and *Dst^Gt^* homozygotes

**Fig. 1.**
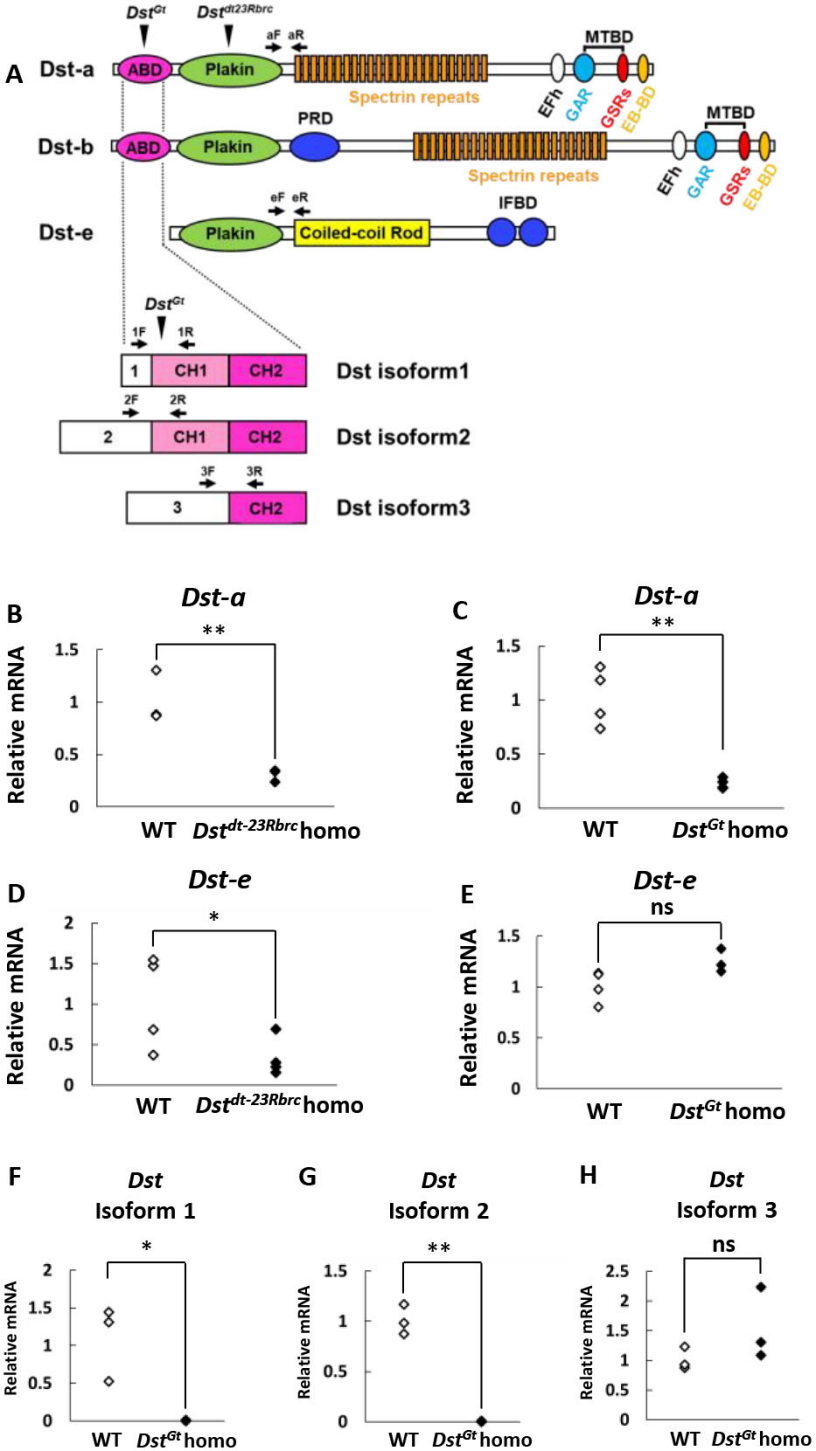
**Expression of *Dst* isoforms in *Dst^dt-23Rbrc^* and *Dst^Gt^* homozygotes.** (A) The structure of the Dst isoforms (Dst-a, Dst-b and Dst-e). Three Dst isoforms are characterized with structural variety in N-terminal regions containing actin-binding domains (ABDs). The ABD of Dst isoforms 1 and 2 consists of two calponin homology domains (CH1 and CH2), and the ABD of Dst isoform 3 contains a single CH2. A gene-trap cassette is inserted within the CH1 of the *Dst^Gt^* allele (*Dst^Gt^* arrowheads). The *Dst^dt-23Rbrc^* allele possesses the nonsense mutation within the plakin domain shared by all Dst isoforms (*Dst^dt-23Rbrc^* arrowhead). EB-BD, EB-binding domain; EFh, EF-hand calcium-binding domain; GAR, growth arrest-specific protein 2-related domain; IFBD, intermediate filament-binding domain; PRD, plakin repeat domain. (B-H) mRNA levels of *Dst* isoforms in samples from *Dst^dt-23Rbrc^* and *Dst^Gt^* homozygotes at 3 weeks of age were analyzed by qPCR. Data were normalized to those of *Actb* (for *Dst-a*, *Dst* isoform 1, *Dst* isoform 2 and *Dst* isoform 3) or those of *Gapdh* (for *Dst-e*). Relative mRNA levels of *Dst-a* and *Dst-e* in the brain (*n*=3) and back skin (*n*=5) of wild type (WT) and *Dst^dt-23Rbrc^* homozygotes (homo) (B,D). mRNA levels of *Dst-a* and *Dst-e* in the brain (*n*=4) and skin (*n*=4 WT, *n*=3 homo) of WT and *Dst^Gt^* homo (C,E). Relative mRNA levels of *Dst* isoforms 1, 2 and 3 in the brain (*n*=3 mice in each genotype) of WT and *Dst^Gt^* homo (F-H). Data are presented as mean±s.d. *P<0.05 and **P<0.01; ns, not statistically significant (*P*>0.05) (Student's *t*-test).

The three major *Dst* isoforms are predominantly expressed in neural, muscular and epidermal tissues (*Dst-a*, *Dst-b* and *Dst-e*), all of which have distinct cytoskeleton-binding domains (Fig. 1A) (Leung et al., 2001; Horie et al., 2017). Each Dst isoform contains various domains, including cytoskeleton-binding domains: plakin repeat domains (PRDs), ABDs composed of calponin homology (CH) domains, coiled-coil rods (CC-ROD), Gly-Ser-Arg (GSR) repeats, EF-hand (EFh) domains, GAS2-related (GAR) domains, and end-binding protein 1 and 3 (EB1/3) domains (Künzli et al., 2016). *Dst-a* and *Dst-e* isoforms are mainly expressed in neural and cutaneous tissues, respectively. Quantitative polymerase chain reaction (qPCR) analysis was performed to quantify the expression levels of *Dst* transcripts in neural and cutaneous tissues from *Dst^dt-23Rbrc^* and *Dst^Gt^* mice. *Dst-a* mRNA from brain extracts were significantly reduced in both *Dst^dt-23Rbrc^* and *Dst^Gt^* homozygous mice relative to wild-type (WT) mice (Fig. 1B,C). Conversely, a significant reduction in *Dst-e* mRNA in skin extracts was observed in *Dst^dt-23Rbrc^* homozygous mice (Fig. 1D) but not in *Dst^Gt^* homozygous mice (Fig. 1E).

In the N-terminus of Dst-a and Dst-b, structural diversity is generated by three different promoters, resulting in six different isoforms, termed Dst-a1, -a2 and -a3 and Dst-b1, -b2 and -b3 (Fig. 1A) (Jefferson et al., 2006). Dst-a1, -a2 and -a3 are suggested to display distinct subcellular localization and functions (Jefferson et al., 2006). In the brain of *Dst^Gt^* mice, there was a remarkable decrease in the expression levels of *Dst* isoforms 1 and 2 compared with those in WT mice, whereas there was no significant change in the expression of *Dst* isoform 3 (Fig. 1F-H). The selective deficiency of *Dst-a1* and *Dst-a2* isoforms in the brains of *Dst^Gt^* homozygous mice was expected because the gene-trap cassette is located downstream of *Dst-a1* and *Dst-a2* transcription initiation sites and upstream of the *Dst-a3* promoter. In the brains of *Dst^dt-23Rbrc^* homozygous mice, there was a decrease in the expression levels of all *Dst* isoforms compared with those in WT (Fig. S1).

### *In situ* hybridization of *Dst* mRNA expression

**Fig. 2.**
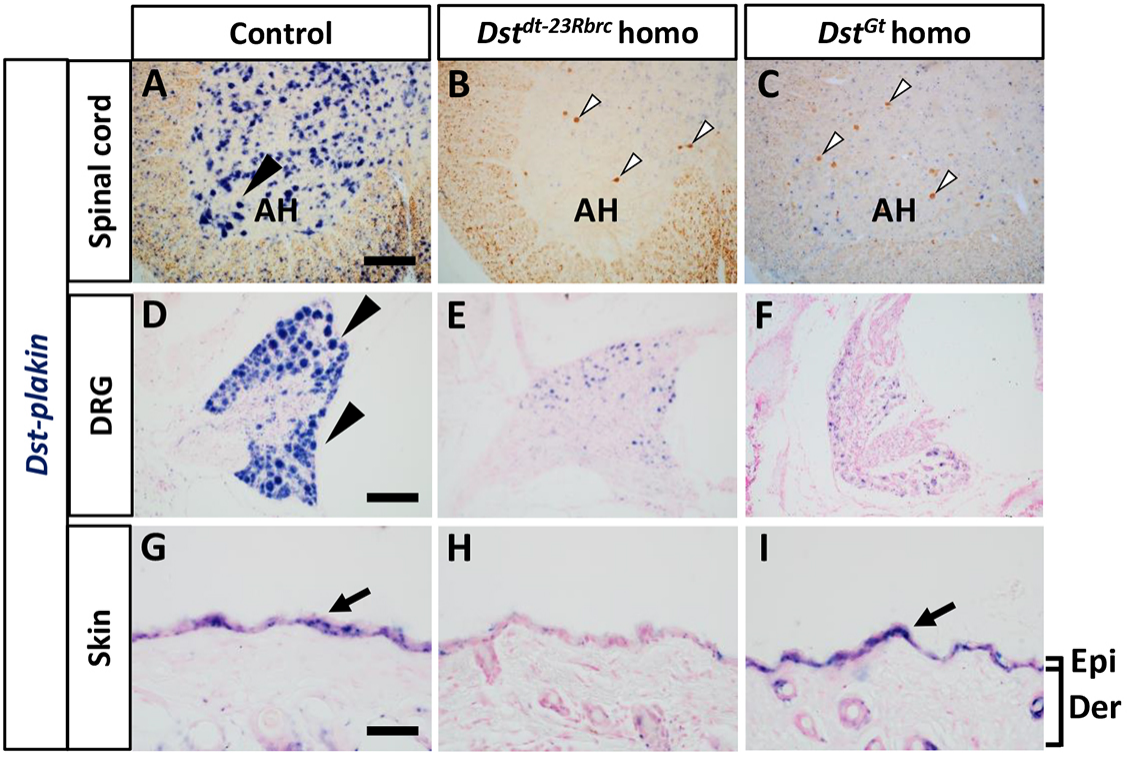
***Dst* mRNA expression in the neural and skin tissues of *Dst^dt-23Rbrc^* and *Dst^Gt^* homozygotes.** (A-I) *In situ* hybridization was performed using *Dst-plakin* probe, which detects all *Dst* isoforms. Distribution of *Dst* mRNA in the spinal cord (A-C), DRG (D-F) and back skin (G-I) of control (A,D,G,I), *Dst^dt-23Rbrc^* homo (B,E,H) and *Dst^Gt^* homo (C,F,I) at 3 weeks (*n*=3 mice in each genotype). In the spinal cord sections (A-C), neurofilament immunostaining was performed after *Dst in situ* hybridization. *Dst* mRNA expression was observed in the control (black arrowheads and arrows in A,D,G,I). Note that *Dst* mRNA expression was decreased in both neural and skin tissues of *Dst^dt-23Rbrc^* mice (B,E,H), but only in neural tissue of *Dst^Gt^* mice (C,F,I). White arrowheads show neurofilament accumulation in the spinal cord of *Dst^dt-23Rbrc^* homo (B) and *Dst^Gt^* homo (C). Scale bars: 120 μm in A and D, 40 μm in G. AH, anterior horn; Der, dermis; Epi, epidermis.

*In situ* hybridization was performed to examine the distribution of *Dst* transcripts in neural and skin tissues. We used a *Dst-plakin* probe, which detects all *Dst* isoforms, for accurately comparing *Dst* expression levels for all isoforms across tissues. In the nervous system of control mice, *Dst* mRNA is strongly expressed in neurons in the spinal cord and sensory neurons in the DRG (Fig. 2A,D). In both *Dst^dt-23Rbrc^* homozygous mice (Fig. 2B,E) and *Dst^Gt^* homozygous mice (Fig. 2C,F), *Dst* expression in these neurons was markedly reduced compared with that in control mice. In the skin of control mice, *Dst* mRNA was detected on epidermal cells (Fig. 2G). *Dst* mRNA was diminished in the epidermis of *Dst^dt-23Rbrc^* homozygous mice (Fig. 2H), but was unaltered in *Dst^Gt^* homozygous mice (Fig. 2I). Data from our *in situ* hybridization analyses are consistent with our qPCR data: *Dst^dt-23Rbrc^* homozygotes display reduced expression in both neural tissue and skin, whereas *Dst^Gt^* homozygotes have reduced expression only in neural tissue, not in the skin. Next, we used a *Dst-SR* probe, which hybridizes with the spectrin repeats of *Dst-a* and *Dst-b* isoforms, but not with those of the *Dst-e* isoform. A similar distribution pattern of *Dst* mRNA was observed in the spinal cord and DRG (Fig. S2A-F). *Dst* mRNA in the epidermis was below detectable levels using the *Dst-SR* probe for all genotypes (Fig. S2G-I).

### Western blot analysis of the Dst proteins

**Fig. 3.**
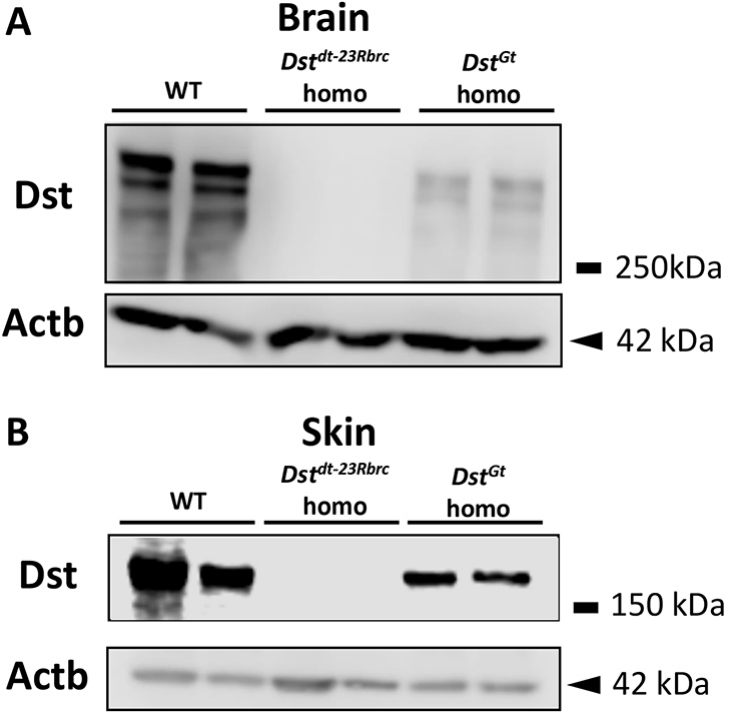
**Dst protein expression in the brain and skin of *Dst^dt-23Rbrc^* and *Dst^Gt^* homozygotes.** (A,B) Western blot analysis using the rabbit polyclonal anti-Dst antibody in brain and skin at 3 weeks (*n*=2 mice in each genotype). Several bands were detected in the brain of WT, which were not detected in that of *Dst^dt-23Rbrc^* homo. The bands were faintly detected in the brain of *Dst^Gt^* homo (A). A single band was detected in the skin of WT and *Dst^Gt^* homo, but was not detected in the skin of *Dst^dt-23Rbrc^* homo (B). β-Actin (Actb, 42 kDa) was used as an internal control.

To detect Dst protein expression in the brain and skin tissue, we performed western blot analysis using a rabbit polyclonal anti-Dst antibody, which recognizes the shared plakin domain. In WT brain extracts, several bands were detected. These bands were not detected in brain extracts of *Dst^dt-23Rbrc^* homozygous mice and only faintly detected in those of *Dst^Gt^* homozygous mice (Fig. 3A). In the skin of WT and *Dst^Gt^* homozygotes, a single Dst-e band was detected. The band was undetectable in the skin of *Dst^dt-23Rbrc^* homozygotes (Fig. 3B).

### Skin pathology in the *Dst* mutants

**Fig. 4.**
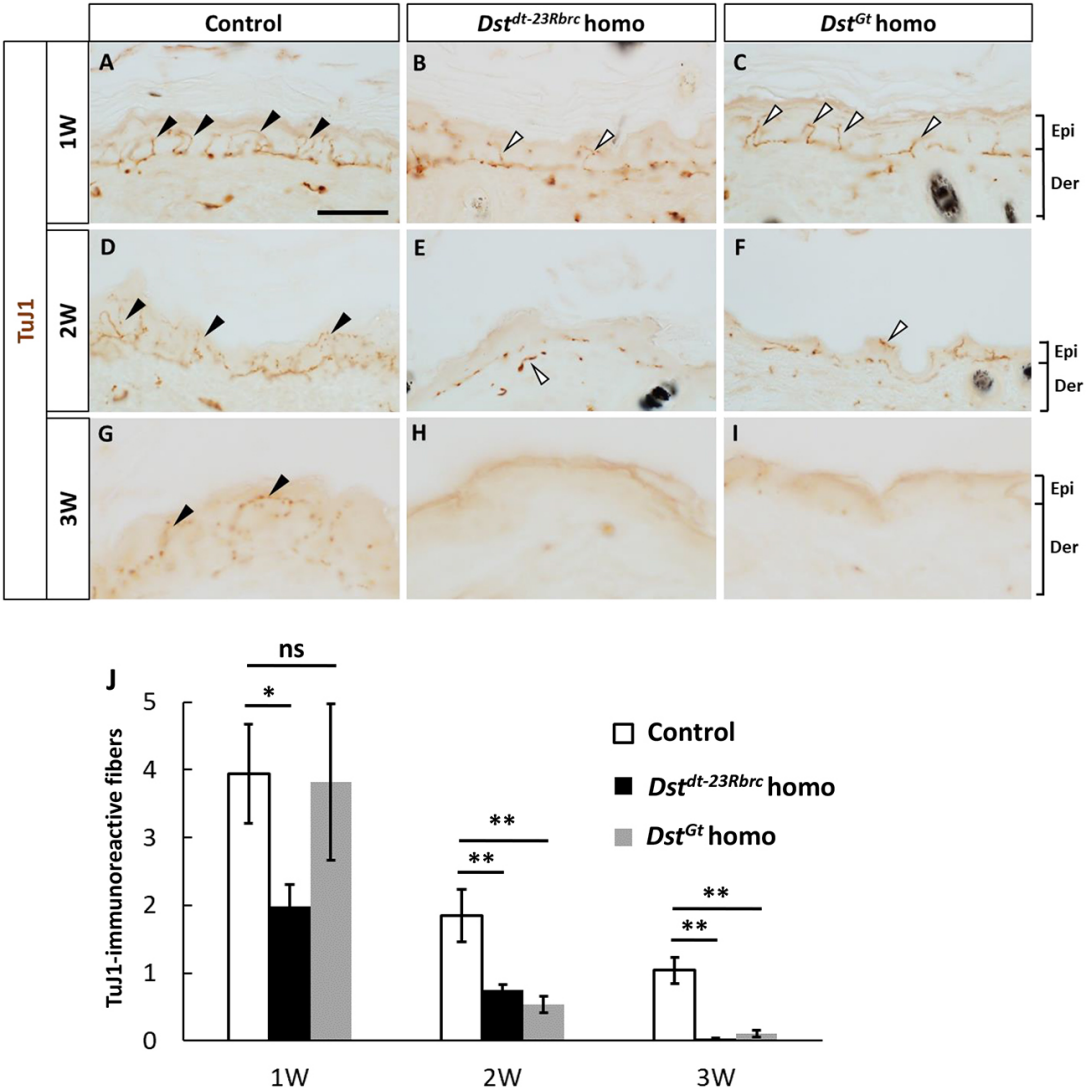
**Peripheral nerve fibers in the skin of *Dst^dt-23Rbrc^* and *Dst^Gt^* homozygotes.** (A-I) Cutaneous innervation of *Dst^dt-23Rbrc^* and *Dst^Gt^* homozygotes was examined at postnatal stages (*n*=3 mice in each genotype). TuJ1 IHC in the skin of control (A,D,G), *Dst^dt-23Rbrc^* homo (B,E,H) and *Dst^Gt^* homo (C,F,I) at 1 week (1W; A-C), 2 weeks (2W; D-F) and 3 weeks (3W; G-I). TuJ1-positive nerve fibers were observed in the epidermis of controls at all stages examined (black arrowheads). In *Dst^dt-23Rbrc^* homo (B) and *Dst^Gt^* homo (C), TuJ1-positive fibers were observed at 1 week (white arrowheads); however, there were fewer TuJ1-positive fibers in the *Dst^dt-23Rbrc^* homozygous epidermis. They gradually decreased and become almost absent by 3 weeks. Scale bar: 20 μm. (J) Quantitative data of TuJ1-positive fibers in cutaneous tissue of *dt* mice. Data are presented as mean±s.e. *P<0.05 and **P<0.01; ns, not statistically significant (*P*>0.05) (one-way ANOVA).

A lack of sensory and autonomic nerve fibers has been observed in the skin of patients with HSAN-VI that harbor mutations involving *DST-a* but not *DST-e* (Manganelli et al., 2017). We previously reported that DRG sensory neurons of *Dst^dt-23Rbrc^* and *Dst^Gt^* homozygous mice undergo neurodegeneration (Horie et al., 2014, 2016). To investigate *dt* skin pathology, cutaneous innervation in postnatal *dt* mice was first assessed by immunohistochemistry (IHC) using anti-class III beta-tubulin (TUBB3) antibody (TuJ1) and anti-PGP9.5 antibody, which stain small nerve fibers in the skin (Lauria et al., 2004). In the control mice, TuJ1- and PGP9.5-immunoreactive sensory nerve fibers were detected in the epidermis (Fig. 4A,D,G; Fig. S3A). In both *Dst^dt-23Rbrc^* and *Dst^Gt^* homozygous mice, TuJ1- and PGP9.5-immunoreactive fibers were almost absent at week 3 (Fig. 4H,I; Fig. S3B,C). TuJ1-immunoreactive nerve fibers were observed at 1 week (Fig. 4B,C); however, there were fewer nerve fibers in *Dst^dt-23Rbrc^* homozygous epidermis (Fig. 4B) than in *Dst^Gt^* homozygous epidermis. Over time, TuJ1-immunoreactive fibers gradually decreased in week 2 in both *Dst^dt-23Rbrc^* and *Dst^Gt^* homozygotes (Fig. 4E,F). Loss of TuJ1-immunoreactive fibers in the epidermis was statistically analyzed by two-way analysis of variance (ANOVA) (genotypes and postnatal stages) and one-way ANOVA (genotypes) in each postnatal stage (Fig. 4J). Two-way ANOVA revealed statistically significant differences between WT and both *dt* mutants (control versus *Dst^dt-23Rbrc^* homo, *P*<0.01; control versus *Dst^Gt^* homo, *P*<0.01), but differences between *Dst^dt-23Rbrc^* homozygotes and *Dst^Gt^* homozygotes were not significant (*P*=0.09). Furthermore, the decrease in nerve fibers was significant between weeks 1 and 2 (*P*<0.01) and weeks 2 and 3 (*P*<0.05). One-way ANOVA at 1 week showed significantly fewer nerve fibers in *Dst^dt-23Rbrc^* homozygotes than in the control (*P*<0.05), whereas no significant difference was observed between the control and *Dst^Gt^* homozygotes (*P*=0.87). Statistically significant differences between the control and both *dt* mutants were observed at weeks 2 and 3 (control versus *Dst^dt-23Rbrc^* homo, *P*<0.01; control versus *Dst^Gt^* homo, *P*<0.01; one-way ANOVA). The autonomic nerve fibers around the sweat glands in the footpad were remarkably decreased in both *Dst^dt-23Rbrc^* and *Dst^Gt^* homozygous mice (Fig. S4B,C) compared with control mice (Fig. S4A) at 3 weeks of age. These results indicate that both sensory and autonomic nerves undergo degeneration leading to skin denervation in *Dst^dt-23Rbrc^* and *Dst^Gt^* homozygous mice.

**Fig. 5.**
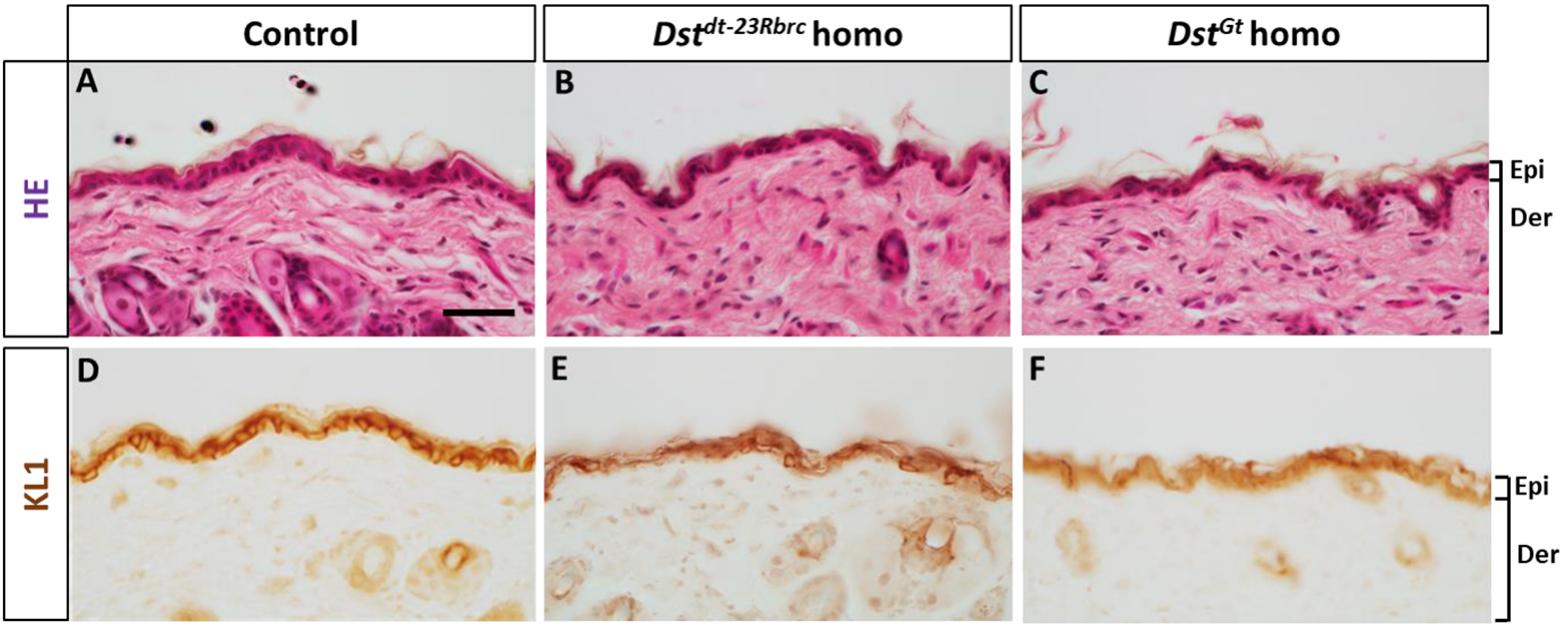
**Light microscopic analyses on the skin of *Dst^dt-23Rbrc^* and *Dst^Gt^* homozygotes.** (A-F) HE and keratin staining in the back skin at 3 weeks (*n*=3 mice in each genotype). HE staining was performed in the skin of control (A), *Dst^dt-23Rbrc^* homo (B) and *Dst^Gt^* homo (C). Keratin IHC was performed using KL1 antibody in the skin of control (D), *Dst^dt-23Rbrc^* homo (E) and *Dst^Gt^* homo (F). The cellular arrangement of the skin was almost normal in the epidermis and dermis in both *Dst^dt-23Rbrc^* homo and *Dst^Gt^* homo. Scale bar: 40 μm.

Because mutations in *DST-e* result in skin fragility and blistering in patients with EBS (Groves et al., 2010), we investigated skin blisters by histological analyses. Hematoxylin and Eosin (HE) staining demonstrated that the morphological arrangement of the back skin was largely intact in *Dst^Gt^* homozygotes and most *Dst^dt-23Rbrc^* homozygotes (Fig. 5A-C). Skin integrity was also investigated by keratin immunostaining (Fig. 5D-F). The frequency of blistering in the skin of *Dst^dt-23Rbrc^* homozygotes was as low as that in the control and not significantly different (control mice, 0.49±0.60%, *n*=3; *Dst^dt-23Rbrc^* homozygotes, 0.82±1.54%, *n*=4; *P*=0.36, Student's *t*-test). These data are consistent with a previous study, which reported that *Dst* knockout mice have less severe blistering in haired skin than in tail skin (Guo et al., 1995). The frequency of blistering in the *Dst^Gt^* homozygous skin was also similar to that in the control (control mice, 0.29±0.50%, *n*=3; *Dst^Gt^* homozygotes, 0.17±0.15%, *n*=3; *P*=0.37, Student's *t*-test).

### Ultrastructure of HDs

**Fig. 6.**
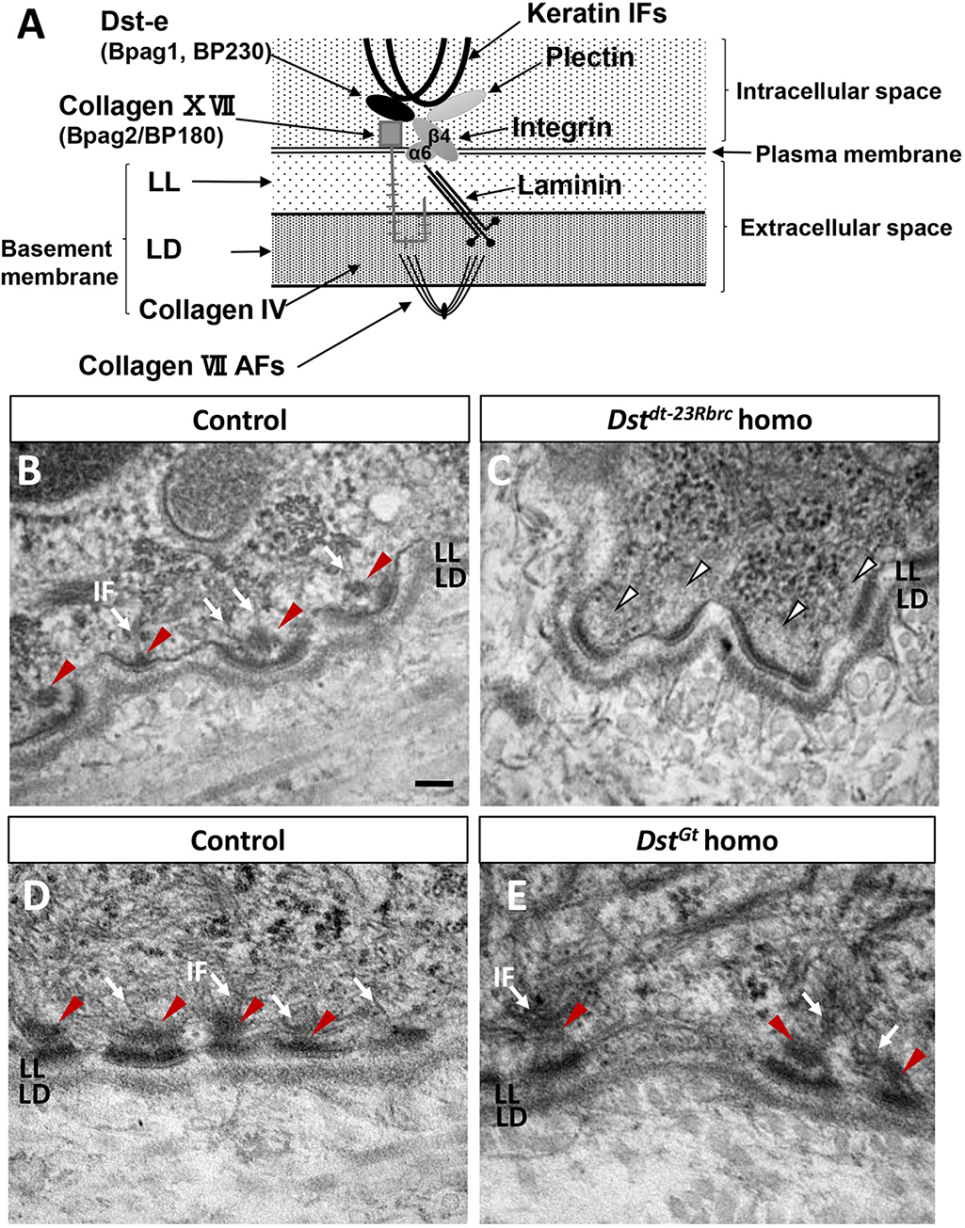
**TEM analyses of the skin of *Dst^dt-23Rbrc^* mice and *Dst^Gt^* mice.** (A) Schematic representation of molecular components in HDs. Dst-e and plectin act as a scaffold in HDs and interact with keratin IF, integrin subunit β4 and collagen XVII. Through such interaction in HDs, keratin networks are linked to the basement membrane. (B-E) TEM in the skin of control (B,D), *Dst^dt-23Rbrc^* homo (C) and *Dst^Gt^* homo (E) at 3 weeks. Inner plaques were observed under HDs of control and *Dst^Gt^* homo (red arrowheads), and in IFs invaded to the inner plaques (arrows). In the skin of *Dst^dt-23Rbrc^* homo, inner plaques and IFs are completely diminished under HDs (white arrowheads). Other structural arrangements of HDs were almost normal in *Dst^dt-23Rbrc^* homo. Scale bar: 100 nm. AF, anchoring fibril; IF, intermediate filament; LD, lamina densa; LL, lamina lucida.

*Dst-e* encodes one of the structural components of HDs, which are adhesion complexes between basal keratinocytes and basement membranes. In HDs, Dst-e localizes to the inner plaque together with plectin (Fig. 6A) (McMillan et al., 2003; Walko et al., 2015). Dst-e and plectin interact with keratin 5/keratin 14 intermediate filaments (IFs), tethering them to the inner plaque of HDs. Dst-e and plectin also form links with transmembrane proteins α6β4 integrin and collagen XVII (Bpag2/BP180; also known as Col17a1) in HDs, with the latter two molecules largely visualized as the outer plaque. In the basement membrane, α6β4 integrin and collagen XVII interact with laminin 332, which extends as thin fibers across the electron-lucent lamina lucida (LL) and finally binds to collagen VII anchoring fibrils (AFs) in the lamina densa (LD). Collagen VII AFs then extend into the dermis (Fig. 6A). Ultrastructure of HDs was visualized using transmission electron microscopy (TEM). Electron-dense cytoplasmic outer and inner plaques were observed in the HDs of control mice (Fig. 6B,D). On the cytoplasmic side of the plasma membrane in the basal keratinocytes, keratin IFs visibly extended to HD inner plaques. In the skin of *Dst^dt-23Rbrc^* homozygous mice, structural abnormalities in HDs were observed (Fig. 6C): the inner plaques of HDs were almost completely absent and the keratin IFs hardly invaded into the HDs, suggesting that the keratin IFs were not anchored underneath the HDs. However, the outer plaque of the HDs and connections across the basement membrane displayed no apparent abnormalities. These abnormalities of HDs in *Dst^dt-23Rbrc^* homozygotes were observed throughout the epidermis. In contrast, *Dst^Gt^* homozygotes displayed no apparent HD abnormalities (Fig. 6E).

## DISCUSSION

In this study, we demonstrated that the expression of *Dst* isoforms is differentially disrupted in *Dst^dt-23Rbrc^* and *Dst^Gt^* mice. In *Dst^dt-23Rbrc^* homozygotes, both *Dst-a* and *Dst-e* transcripts were reduced; probably because of nonsense-mediated mRNA decay induced by a newly introduced stop codon (Figs 1 and 2; Figs S1 and S2). Nonsense mutation in the *Dst^dt-23Rbrc^* allele results in a drastic reduction in Dst protein to below detectable levels in both neural and cutaneous tissues (Fig. 3). In *Dst^Gt^* homozygotes, *Dst-a1* and *Dst-a2* transcripts are reduced, whereas *Dst-a3* in the nervous system and *Dst-e* in the skin are unaffected (Figs 1 and 2; Fig. S1). The expression patterns of *Dst* isoforms account for the residual Dst protein expression patterns in *Dst* mutants (Fig. 3). TEM analyses of the skin revealed that only *Dst^dt-23Rbrc^* homozygotes show a loss of keratin filament contacts underneath the HDs, and that there is no such abnormality in the skin of *Dst^Gt^* homozygotes (Fig. 6). Our data suggest that Dst-e plays a critical role in HD integrity and that *Dst^dt-23Rbrc^* mice are an excellent model for investigating disease mechanisms, not only for HSAN-VI but also for EBS. Our data describe important aspects of phenotypic heterogeneity in *DST*-related diseases.

### Structure and function of Dst-a and Dst-b

The structures of Dst-a and Dst-b are quite similar, and are considered to function as cytoskeletal regulators (Dalpé et al., 1998; Ryan et al., 2012). Indeed, neurons derived from HSAN-VI patient induced pluripotent stem cells have abnormal morphologies, such as short neurites (Manganelli et al., 2017). In addition to the regulation of cell shape, intracellular functions of Dst have also been described; *Dst* knockdown with siRNA impairs vesicular transport and maintenance of the Golgi apparatus (Ryan et al., 2012; Poliakova et al., 2014). Such traffic functions are essential for neurons, including neurite outgrowth, axonal transport and synaptic transmission. Among the domains in Dst-a and Dst-b, a tubulin-binding domain in the C-terminus is critical because the C-terminal truncated mutations occur in patients with HSAN-VI (Edvardson et al., 2012). The ABD at the N-terminus of Dst-a and Dst-b is also critical because a point mutation allele has been reported in patients with HSAN-VI (Fortugno et al., 2019). Although the role of Dst-a in neuronal function has been well investigated, it remains an open question as to how much the *Dst-b* isoform in skeletal and heart muscles contributes to *dt* phenotypes of mutant mice (Horie et al., 2016). Therefore, we have generated and are analyzing *Dst-b*-specific mutant mice.

### *Dst* isoforms and neural phenotypes

Disturbance of sensory and autonomic nervous systems has been commonly reported in patients with HSAN-VI (Edvardson et al., 2012; Manganelli et al., 2017; Fortugno et al., 2019). In both *Dst^dt-23Rbrc^* and *Dst^Gt^* homozygotes, neurodegeneration of sensory and autonomic nervous systems is observed (Fig. 4; Fig. S4). Degeneration of sensory neurons in the DRG has been most frequently described for *dt* mice (Duchen et al., 1964; Horie et al., 2014), suggesting that sensory functions are severely disrupted in *dt* mice. Disturbance of the autonomic nervous system is also a possible factor that alters the life span and phenotypic severity (Tseng et al., 2011). In addition, we assume that abnormal mastication accompanied by the degeneration of motor neurons in the trigeminal motor nucleus is another factor altering systemic conditions in *dt* mice ([Bibr DMM041608C21][Bibr DMM041608C22]).

There is variation in the severity and onset of HSAN-VI manifestations: late-onset patients with HSAN-VI carrying mutations that selectively disrupt *DST-a2* have been reported (Manganelli et al., 2017), whereas the first-described HSAN-VI family harboring frameshift mutations affecting all *DST-a* isoforms displayed more severe phenotypes, including infant death (Edvardson et al., 2012). In *Dst^Gt^* homozygotes, *Dst* isoforms 1 and 2, but not isoform 3, are disrupted by gene-trap insertion in the genomic region encoding the ABD, whereas all *Dst* isoforms have a nonsense mutation in *Dst^dt-23Rbrc^* homozygotes (Fig. 1; Fig. S1). In our observations, sensory denervation in the skin of *Dst^dt-23Rbrc^* homozygotes appeared to occur earlier than that in the skin of *Dst^Gt^* homozygotes (Fig. 4). Although functional differences between all three N-terminal *Dst* isoforms are not fully understood, our histological observations are consistent with a recent study suggesting that *Dst-a3* plays a compensatory function in the neural phenotypes of *dt^Tg4^* homozygous mice, which have defects in *Dst-a1* and *Dst-a2*, but still express *Dst-a3* (Lynch-Godrei et al., 2018). Because transgenic restoration of *Dst-a2* in *dt* mice ameliorates the degeneration of sensory neurons in the DRG and partially extends the life span (Ferrier et al., 2014), *DST-a2* seems to be the most crucial isoform for neurological symptoms in HSAN-VI, whereas the other *DST-a* isoforms may have redundant roles. Although *Dst^Gt^* homozygotes and *Dst^dt-23Rbrc^* homozygotes show almost the same motor abnormality, we previously reported that the dystonic score of *Dst^dt-23Rbrc^* homozygotes is slightly lower than that of *Dst^Gt^* homozygotes (Horie et al., 2016). This observation suggests that factor(s) other than *Dst-a3* isoform expression also affect the *dt* phenotype. One possible factor is genetic background. We used mutant lines with different mouse backgrounds: *Dst^dt-23Rbrc^* homozygotes were on a mixed background of C57BL/6 and C3H/HeN, and *Dst^Gt^* homozygotes were on a C57BL6 background. It is known that mouse background can affect the mutant phenotypes (Olds-Clarke and McCabe, 1982; Lenk and Meisler, 2014; Terumitsu-Tsujita et al., 2019).

### *Dst-e* mutations and skin phenotype

Mutations in *DST-e* result in EBS with skin fragility and blistering (Groves et al., 2010; McGrath, 2015). In the first reported cases of EBS linked to *DST* mutations, the defects were limited to nonsense mutations in the CC-ROD, which is a DST-e-specific domain (Groves et al., 2010; Liu et al., 2012). These patients experienced generalized trauma-induced spontaneous skin blisters. Electron microscopy analyses of both mutations identified a lack of HD inner plaques. Keratin filaments extend to where the inner plaque should be, as though connected, but the presence of connection points is not apparent. Keratinocytes cultured from patients with EBS have adhesion and migration defects (Michael et al., 2014). Patients with EBS have fragile skin due to *DST-e* mutations; mild physical trauma, such as rubbing or scratching, and defects in wound healing processes cause skin blisters. In our analyses, Dst-e protein levels were undetectable in the skin of *Dst^dt-23Rbrc^* mice (Fig. 3); blisters were rarely observed (Fig. 5). One possible reason for the lack of skin blistering in mutant mice is that the short lifespan of *dt* mice hampers long-term analysis. *Dst-e*-specific mutant mice should make it possible to analyze the mechanism of skin blistering in these mice. Because *Dst* knockout mice show a delay in cutaneous wound healing (Guo et al., 1995), it would be interesting to investigate the delay in wound healing in *Dst^dt-23Rbrc^* mice in future studies. We also expect that *dt-MP* (deletion of exon 39 to intron 61; Seehusen et al., 2016) will have a skin phenotype (ultrastructural abnormality in HDs), as well as a neural phenotype, because they disrupt all *Dst-a*, *Dst-b* and *Dst-e* isoforms. The genes other than *Dst-e* that cause EBS are keratin 14 (Bonifas et al., 1991; Coulombe et al., 1991), keratin 5 (Lane et al., 1992) and plectin (Chavanas et al., 1996; Gache et al., 1996; McLean et al., 1996). Plectin (*PLEC*) encodes huge intermediate filament-binding protein and its mutation leads to EBS with muscular dystrophy (Chavanas et al., 1996; Gache et al., 1996; McLean et al., 1996). Conditional deletion of plectin in epidermal cells causes skin blistering in mice (Ackerl et al., 2007). By comparing the severity of EBS manifestation with their causative mutations, one should be able to identify genes and proteins that contribute to maintaining skin strength and integrity.

To understand the pathogenesis of HSAN-VI and EBS, it is important to identify gene mutations in patients with HSAN-VI, patients with EBS and various *dt* mutant models, and to determine their precise phenotypes. It may be useful to check for the existence of HD abnormalities in patients with HSAN-VI and *dt* mutants by TEM analyses to diagnose cutaneous manifestations, and to restrict the genomic region needed for accurate mutation analyses. In addition, we believe that conditional knockout and rescue experiments using the multifunctional *Dst^Gt^* allele will be useful for identifying the neural circuits responsible for movement disorders, and organs or cell types involved in the systemic manifestations in *dt* mice, paving the way for a viable treatment of HSAN-VI.

## MATERIALS AND METHODS

### Animals

Animal care and experimental protocols were approved by the Animal Experiment Committee of Niigata University and were carried out in accordance with the Guidelines for the Care and Use of Laboratory Animals of Niigata University (approved number SA00521). Mice were maintained at 23±3°C, 50±10% humidity, 12 h light/dark cycles and food/water availability *ad libitum*. *Dst^dt-23Rbrc^* mice [RBRC01615, Mouse Genome Informatics (MGI) number: 6119708] (Horie et al., 2016) and *Dst^Gt(E182H05)^* mice (MGI: 3917429) (Horie et al., 2014) were used in this study. The *Dst^Gt(E182H05)^* allele was abbreviated as *Dst^Gt^*. Homozygous *Dst^dt-23Rbrc^* and *Dst^Gt^* mice were obtained by heterozygous mating. The day of birth was recorded as postnatal day 0 (P0). Genotyping PCR for the *Dst^Gt^* allele was performed as previously described (Horie et al., 2014). For genotyping PCR of the *Dst^dt-23Rbrc^* allele, we used an improved PCR primer set, wherein the MnlI site within the primer sequence was disrupted as follows: dt23F3 primer, 5′-CCTGGCTATGCTCCAGGAAAT-3′ and dt23R3 primer, 5′-GCCACGCCATTAATCCAAGG-3′. PCR conditions and restriction fragment length polymorphism protocols were as previously described (Horie et al., 2016).

### RNA extraction and real-time PCR

qPCR was performed as previously described (Hayakawa-Yano et al., 2017). Total RNA was extracted from the brain and back skin using an RNeasy Mini Kit (Qiagen, Hilden, Germany). One microgram of RNA template was used for cDNA synthesis with oligo (dT) primers. Real-time PCR was performed using a StepOnePlus Real-Time PCR system (Thermo Fisher Scientific, Waltham, MA, USA) and the following cycling conditions: 95°C for 2 min, followed by 40 cycles of 95°C for 15 s, 60°C for 40 s and 95°C for 15 s. Expression levels of *Dst-a*, *Dst-e* and *Dst* isoforms 1, 2, and 3 were analyzed using the ΔΔCT method. β-Actin (*Actb*) and glyceraldehyde-3-phosphate dehydrogenase (*Gapdh*) were used as internal controls to normalize the variability of expression levels for *Dst-a* and *Dst-e*. For semi-quantitative reverse-transcription PCR, the PCR reaction was performed using the same cDNA samples with the following cycling conditions: 94°C for 2 min, followed by 27-30 cycles of 94°C for 30 s, 58°C for 30 s and 65°C for 30 s. The following primers were used: *Dst-a* forward, 5′-AACCCTCAGGAGAGTCGAAGGT-3′ and *Dst-a* reverse, 5′-TGCCGTCTCCAATCACAAAG-3′; *Dst-e* forward, 5′-TGAGAATAGCAAACTTAGCGGGA-3′ and *Dst-e* reverse, 5′-CGGCCTCCTTAACTTTCGG-3′; *Dst* isoform 1 forward, 5′-TCCAGGCCTATGAGGATGTC-3′ and *Dst* isoform 1 reverse, 5′-GGAGGGAGATCAAATTGTGC-3′; *Dst* isoform 2 forward, 5′-AATTTGCCCAAGCATGAGAG-3′ and *Dst* isoform 2 reverse, 5′-CGTCCCTCAGATCCTCGTAG-3′; *Dst* isoform 3 forward, 5′-CACCGTCTTCAGCTCACAAA-3′ and *Dst* isoform 3 reverse, 5′-AGTTTCCCATCTCTCCAGCA-3′; *Actb* forward, 5′-GGCTGTATTCCCCTCCATCG-3′ and *Actb* reverse, 5′-CCAGTTGGTAACAATGCCATGT-3′; *Gapdh* forward, 5′-AGGTCGGTGTGAACGGATTTG-3′ and *Gapdh* reverse, 5′-TGTAGACCATGTAGTTGAGGTCA-3′.

### Histological procedures

For tissue preparations, mice were euthanized via intraperitoneal injection with pentobarbital sodium (100 mg/kg body weight), and then perfused with 4% paraformaldehyde (PFA) in 0.1 M phosphate-buffered solution (PB) (pH 7.4). The tissues were fixed by cardiac perfusion with 0.01 M phosphate-buffered saline (PBS) followed by ice-cold 4% PFA in 0.1 M PB (pH 7.4). Dissected tissues were immersed in the same fixative overnight. To cut spinal cord and DRG sections, the specimens were rinsed with water for 10 min and decalcified in Morse solution (135-17071; Wako, Osaka, Japan) overnight. Tissues were then dehydrated using an ascending series of ethanol and xylene washes, and embedded in paraffin (P3683; Paraplast Plus; Sigma-Aldrich, St Louis, MO, USA). Consecutive 10-μm-thick paraffin sections were cut on a rotary microtome (HM325; Thermo Fisher Scientific), mounted on MAS-coated glass slides (Matsunami Glass, Osaka, Japan) and air-dried on a hot plate overnight at 37°C. Paraffin sections were deparaffinized in xylene, rehydrated using a descending series of ethanol washes, and then rinsed in distilled water. For HE staining, sections were stained with Mayer's Hematoxylin (131-09665; Wako) for 10 min and with 0.2% Eosin Y in ethanol for 5 min.

For IHC, deparaffinized sections were treated with microwave irradiation in 10 mM citric acid buffer, pH 6.0, for 5 min, and incubated overnight at 4°C with the following primary antibodies: rabbit polyclonal anti-PGP9.5/ubiquitin carboxy-terminal hydrolase L1 (UCHL1) antibody (1:1000; UltraClone, Isle of Wight, UK, purchased from Cosmo Bio Inc., Tokyo, Japan), mouse monoclonal anti-tubulin β3 (TUBB3) antibody (1:2000; RRID:AB_10063408, clone TuJ1; BioLegend, San Diego, CA, USA), mouse anti-keratin antibody (1:100; KL1; Immunotech, Marseille, France) and mouse monoclonal anti-neurofilament-M (NF-M) antibody (1:500; 1C8; Watanabe et al., 2006), all diluted in 0.1 M PBS with 0.01% Triton X-100 (PBST) containing 0.5% skim milk. Sections were then incubated in horseradish peroxidase-conjugated secondary antibody (1:200; MBL, Nagoya, Japan) diluted in PBST containing 0.5% skim milk for 60 min at 37°C. Between each step, sections were rinsed in PBST for 15 min. After rinsing sections in distilled water, immunoreactivity was visualized in 50 mM Tris-HCl buffer (pH 7.4) containing 0.01% diaminobenzidine tetrahydrochloride and 0.01% hydrogen peroxide at 37°C for 5 min. Sections were then dehydrated through graded ethanols and xylene solutions and placed on coverslips with Bioleit (23-1002; Okenshoji, Tokyo, Japan). Digital images were taken with a microscope (BX53; Olympus, Tokyo, Japan) equipped with a digital camera (DP74, Olympus), and the TIF files were processed with Photoshop software (Adobe, San Jose, CA, USA).

### *In situ* hybridization

*In situ* hybridization was performed on paraffin sections as described in previous studies (Takebayashi et al., 2000; Hossain et al., 2018a,b). *Dst-plakin* probe [GenBank accession number NM_001276764, nucleotides (nt) 2185-3396] and *Dst-SR* probe (GenBank accession number NM_001276764, nt 15994-17059; Horie et al., 2014) were used. The *Dst-plakin* probe recognizes *Dst-a*, *Dst-b* and *Dst-e* isoforms and the *Dst-SR* probe recognizes only *Dst-a* and *Dst-b* isoforms. After staining with *Dst-plakin* probe, spinal cord sections were subsequently immunostained with anti-NF-M antibody and other sections (DRG and back skin) were counterstained by Nuclear Fast Red.

### Western blotting

Western blotting was performed as previously described (Zhou et al., 2018). Frozen brain and skin tissues were homogenized using a Teflon-glass homogenizer in ice-cold homogenization buffer (0.32 M sucrose, 5 mM EDTA, 10 mM Tris-HCl, pH 7.4, phosphatase inhibitor cocktail tablet; Roche, Mannheim, Germany), centrifuged at 1900 ***g*** for 10 min at 4°C and the supernatants were collected. The protein concentration was determined using bicinchoninic acid protein assay reagent (Thermo Fisher Scientific). Lysates were mixed with an equal volume of 2× sodium dodecyl sulfate (SDS) sample buffer (125 mM Tris-HCl, pH 6.8, 4% SDS, 20% glycerol and 0.002% Bromophenol Blue) for a final protein concentration of 1.5-2 μg/μl and denatured in the presence of 100 mM dithiothreitol at 100°C for 5 min. SDS-polyacrylamide gel electrophoresis (PAGE) was performed with 20 μg per lane for brain samples or 30 μg per lane for skin samples on 5-20% gradient gels (197-15011, SuperSep™ Ace; FUJIFILM Wako Pure Chemical Corporation, Osaka, Japan) running at 10-20 mA for 150 min. The gels were blotted onto an Immobilon-P transfer membrane (Millipore, Billerica, MA, USA). After blocking with 10% skim milk for 3 h, blotted membranes were incubated with the following primary antibodies: rabbit polyclonal anti-Dst antibody (gifted from Dr Ronald K Leim; Goryunov et al., 2007), which recognizes the plakin domain of Dst, and mouse monoclonal anti-Actb antibody (1:2000; AB_2223041, clone C4; Merck Millipore). Each primary antibody was incubated overnight at 4°C. Then membranes were incubated with peroxidase-conjugated secondary antibodies for 1 h at room temperature: anti-rabbit immunoglobulin G (IgG) (1:2000; AB_2099233, Cat. #7074; Cell Signaling Technology, Beverly, MA, USA), anti-mouse IgG (1:2000; AB_330924, Cat. #7076; Cell Signaling Technology). Tris-buffered saline (10 mM Tris-HCl, pH 7.5, 150 mM NaCl) containing 0.1% Tween-20 and 10% skim milk was used for the dilution of primary and secondary antibodies, and Tris-buffered saline containing 0.1% Tween-20 was used as the washing buffer. Immunoreactions were visualized by ECL (GE Healthcare, Piscataway Township, NJ, USA) and a luminescence image analyzer (C-Digit; LI-COR, Lincoln, NE, USA).

### TEM

For TEM analysis, tissue preparation was performed as previously described (Eady, 1985). Tissue blocks (back skin and foot sole skin) were fixed using 2.5% glutaraldehyde in 0.1 M PB (pH 7.4), cut into small pieces (∼3×3 mm), and further fixed in the same fixative over 1 day at 4°C. Next, samples were fixed with 1% osmium tetroxide in 0.1 M PB, dehydrated using a graded series of ethanol washes, transferred into propylene oxide and embedded in epoxy resin (Epon 812; TAAB, Berkshire, UK) for polymerization over 24 h at 60°C. Ultrathin sections with a thickness of 70 nm were prepared using an ultramicrotome (Ultracut N; Reichert-Nissei, Tokyo, Japan), stained with 1% uranyl acetate for 10 min, followed by 1% lead citrate for 5 min (Richardson et al., 1960), and observed under a TEM (H-7650; Hitachi, Tokyo, Japan) at an accelerating voltage of 80 kV.

### Quantification and statistical analysis

To quantify qPCR data, Student's *t*-test was performed. Quantification of TuJ1-immunoreactive fibers was performed with MetaMorph software (Meta Series Software ver. 7.10.2; Molecular Devices, San Jose, CA, USA). The area of TuJ1-immunoreactive fibers within the epidermis was normalized by the surface length of the skin sections. Morphometric analysis was performed on three sections per mouse, with three or more mice per group. For statistical analysis, one-way and two-way ANOVA were performed to compare controls, *Dst^dt-23Rbrc^* homozygotes and *Dst^Gt^* homozygotes across postnatal 1, 2 and 3 weeks. Statistical analysis was performed using ANOVA4 on the Web (https://www.hju.ac.jp/~kiriki/anova4/).

## Supplementary Material

Supplementary information
