# Supplementary material for: Diverse dystonin gene mutations cause distinct patterns of Dst isoform deficiency and phenotypic heterogeneity in Dystonia musculorum mice
Source: Dis Model Mech. 2020 May 21;13(5):dmm041608. doi: 10.1242/dmm.041608 (PMC7325434; doi:10.1242/dmm.041608)
Supplement: Supplementary information [file dmm-13-041608-s1.pdf]

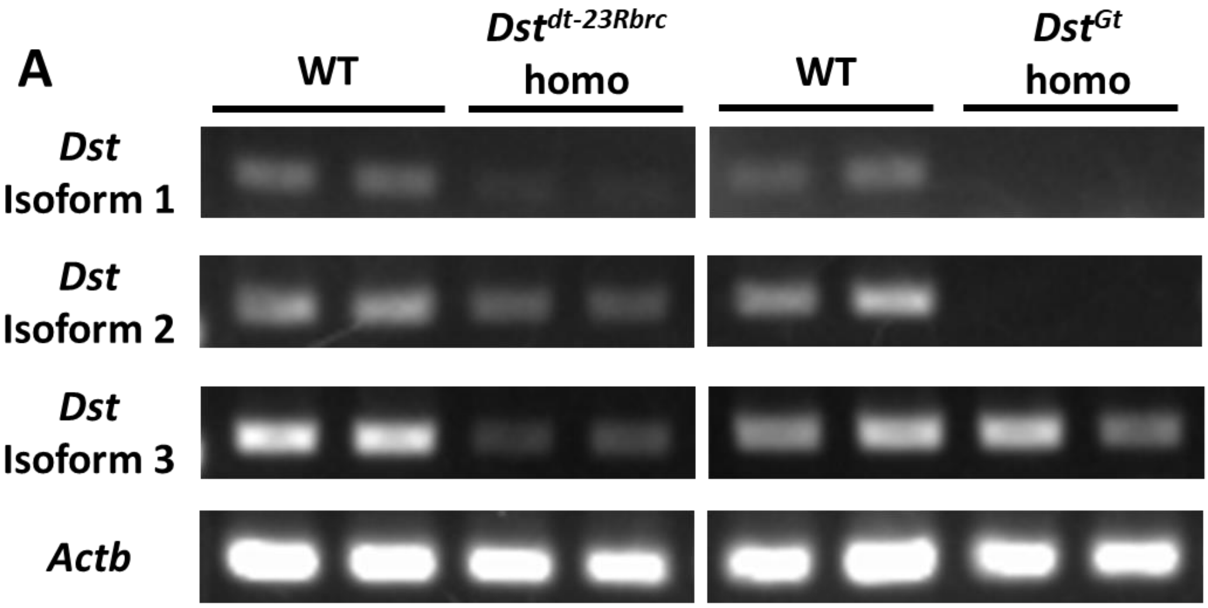

Fig. S1. Expression of *Dst* isoforms in the brain of $Dst^{dt\text{-}23Rbrc}$ mice and Dst^{Gt} mice.

Detection of the *Dst* isoforms (isoform 1, isoform 2 and isoform 3) in the brain of $Dst^{dt\text{-}23Rbrc}$ mice (left) and Dst^{Gt} mice (right) at 3 weeks old. In wild-type mice, all *Dst* isoforms were detected. In $Dst^{dt\text{-}23Rbrc}$ homozygotes, reduced expressions of all *Dst* isoforms were observed. In Dst^{Gt} homozygotes, *Dst* isoform1 and *Dst* isoform 2 were undetectable, whereas expression of *Dst* isoform 3 was almost comparable to WT. *Actb* was used as an internal control.

Disease Models & Mechanisms • Supplementary information

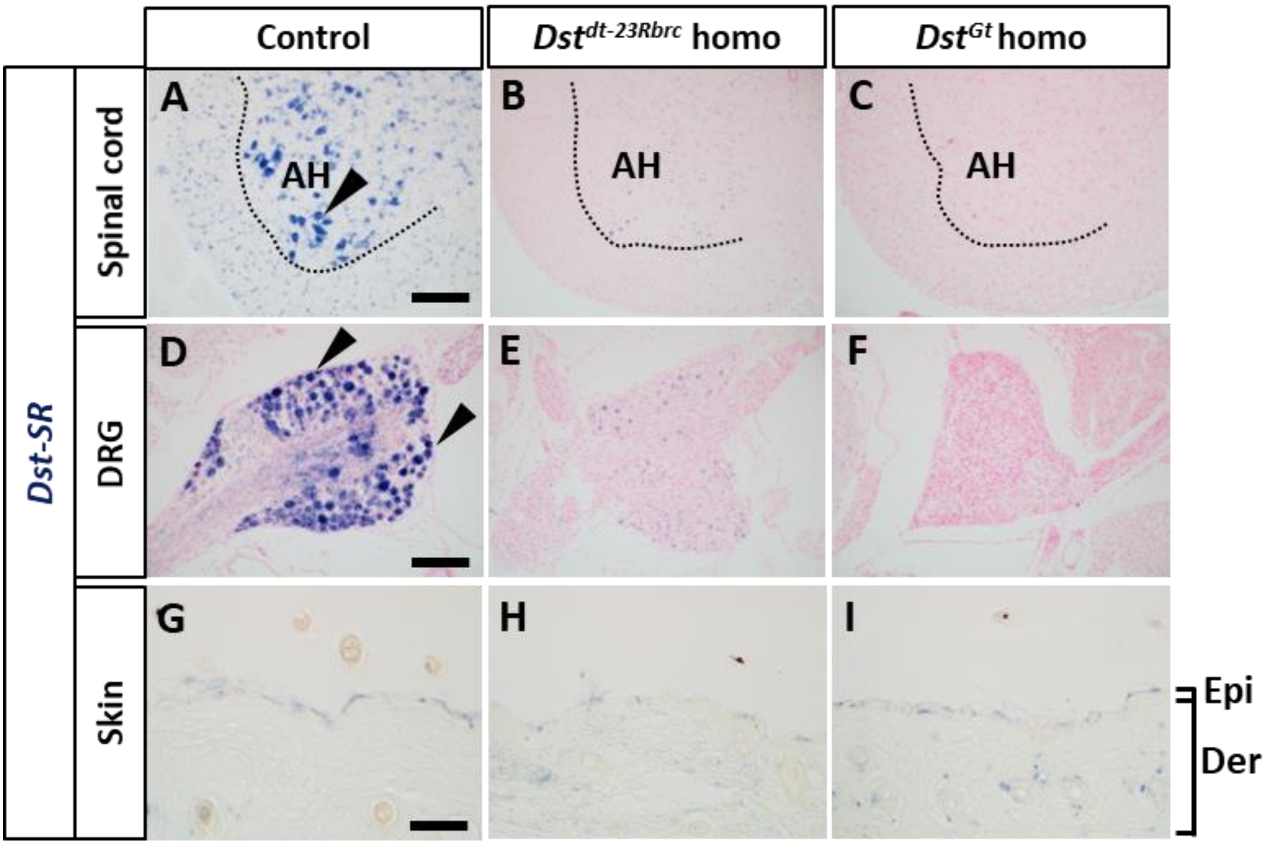

Fig. S2. *Dst* distribution in the neural and cutaneous tissues of *Dst*$^{dt\text{-}23Rbrc}$ and *Dst*^{Gt} mice.

(A-I) *In situ* hybridization was performed using *Dst-SR* probe, which detects *Dst-a* and *Dst-b* but not *Dst-e*. *Dst* mRNA in the spinal cord (A-C), DRG (D-F) and skin (G-I) of control (A, D and G), *Dst*$^{dt\text{-}23Rbrc}$ homo (B, E and H) and *Dst*^{Gt} homo (C, F and I) at 3 weeks. *Dst* mRNA expression was observed in the neural tissues of control mice (arrowheads in A, D). *Dst* mRNA was decreased in neural tissue of *Dst*$^{dt\text{-}23Rbrc}$ and *Dst*^{Gt} homo mice. *Dst* mRNA in the skin was under detectable level by the *Dst-SR* probe. Scale bars, 120 µm in A (applied to A-C) and 40 µm in D (applied to D-F). Dotted line indicated the border between gray and white matters. AH: anterior horn, Epi: epidermis, Der: dermis.

Disease Models & Mechanisms • Supplementary information

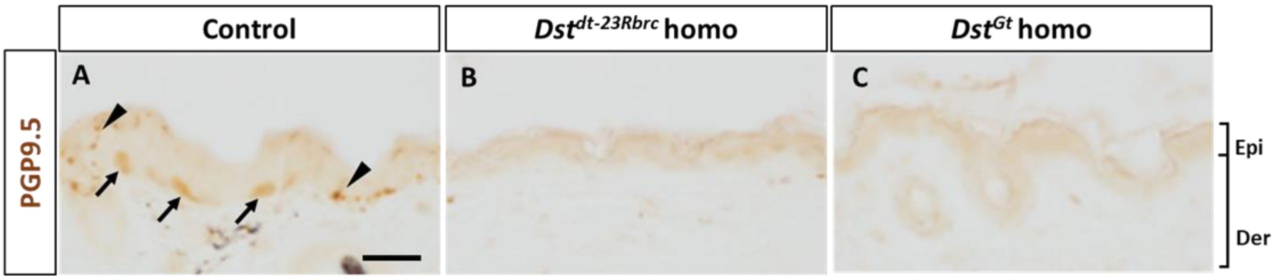

Fig. S3. Peripheral nerve fibers in the skin of *Dst*$^{dt\text{-}23Rbrc}$ and *Dst*^{Gt} homozygotes.

(A-C) Cutaneous innervation of *dt* mice at 3 weeks. PGP9.5 IHC was performed in the skin of control (A), *Dst*$^{dt\text{-}23Rbrc}$ homo (B) and *Dst*^{Gt} homo (C). Small nerve fibers (arrowheads) and large nerves (arrows) were observed in control (A). *Dst*$^{dt\text{-}23Rbrc}$ homo (B) and *Dst*^{Gt} homo (C) showed the loss of sensory nerve fibers in the epidermis. Scale bars, 20 µm.

Disease Models & Mechanisms • Supplementary information

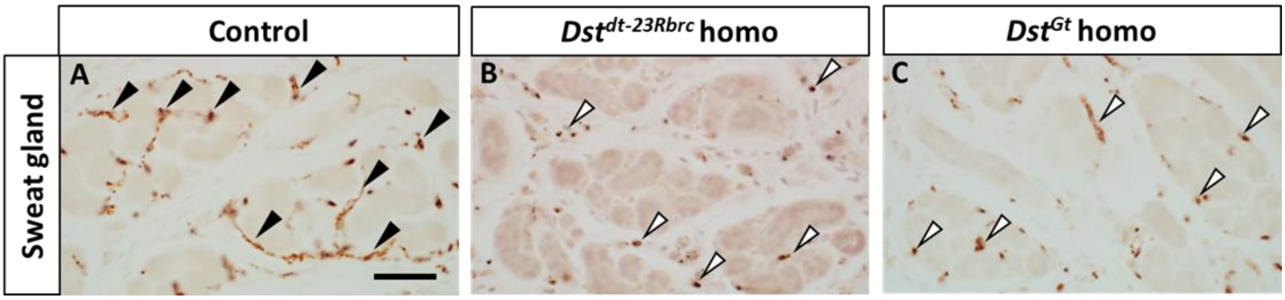

Fig. S4. Autonomic nerve fibers in the sweat gland of *dt* mice.

(A-C) Autonomic nerve fibers in *dt* mice at 3 weeks. TuJ1 IHC in control (A), *Dst*$^{dt\text{-}23Rbrc}$ homo (B) and *Dst*^{Gt} homo (C) showed many nerve fibers around sweat gland in the footpad (arrowheads in A) were observed in control, while very few nerve fibers were visible in *Dst*$^{dt\text{-}23Rbrc}$ homo (white arrowheads in B) and *Dst*^{Gt} homo (white arrowheads in C). Scale bars, 40 µm in A (applied to A-C).

Disease Models & Mechanisms • Supplementary information
